# Molecular Breeding Strategy and Challenges Towards Improvement of Blast Disease Resistance in Rice Crop

**DOI:** 10.3389/fpls.2015.00886

**Published:** 2015-11-16

**Authors:** Sadegh Ashkani, Mohd Y. Rafii, Mahmoodreza Shabanimofrad, Gous Miah, Mahbod Sahebi, Parisa Azizi, Fatah A. Tanweer, Mohd Sayeed Akhtar, Abbas Nasehi

**Affiliations:** ^1^Laboratory of Food Crops, Institute of Tropical Agriculture, Universiti Putra MalaysiaSerdang, Malaysia; ^2^Department of Agronomy and Plant Breeding, Yadegar –e- Imam Khomeini RAH Shahre-Rey Branch, Islamic Azad UniversityTehran, Iran; ^3^Department of Crop Science, Faculty of Agriculture, Universiti Putra MalaysiaSerdang, Malaysia; ^4^Laboratory of Plantation Crops, Institute of Tropical Agriculture, Universiti Putra MalaysiaSerdang, Malaysia; ^5^Department of Plant Breeding and Genetics, Faculty of Crop Production, Sindh Agriculture University TandojamSindh, Pakistan; ^6^Department of Botany, Gandhi Faiz-e-Aam CollegeShahjahanpur, India; ^7^Department of Plant Protection, Faculty of Agriculture, Universiti Putra MalaysiaSerdang, Malaysia

**Keywords:** rice blast disease, molecular breeding, DNA markers, QTL mapping, marker-aided selection, gene pyramiding

## Abstract

Rice is a staple and most important security food crop consumed by almost half of the world’s population. More rice production is needed due to the rapid population growth in the world. Rice blast caused by the fungus, *Magnaporthe oryzae* is one of the most destructive diseases of this crop in different part of the world. Breakdown of blast resistance is the major cause of yield instability in several rice growing areas. There is a need to develop strategies providing long-lasting disease resistance against a broad spectrum of pathogens, giving protection for a long time over a broad geographic area, promising for sustainable rice production in the future. So far, molecular breeding approaches involving DNA markers, such as QTL mapping, marker-aided selection, gene pyramiding, allele mining and genetic transformation have been used to develop new resistant rice cultivars. Such techniques now are used as a low-cost, high-throughput alternative to conventional methods allowing rapid introgression of disease resistance genes into susceptible varieties as well as the incorporation of multiple genes into individual lines for more durable blast resistance. The paper briefly reviewed the progress of studies on this aspect to provide the interest information for rice disease resistance breeding. This review includes examples of how advanced molecular method have been used in breeding programs for improving blast resistance. New information and knowledge gained from previous research on the recent strategy and challenges towards improvement of blast disease such as pyramiding disease resistance gene for creating new rice varieties with high resistance against multiple diseases will undoubtedly provide new insights into the rice disease control.

## Introduction

**FIGURE 1:**
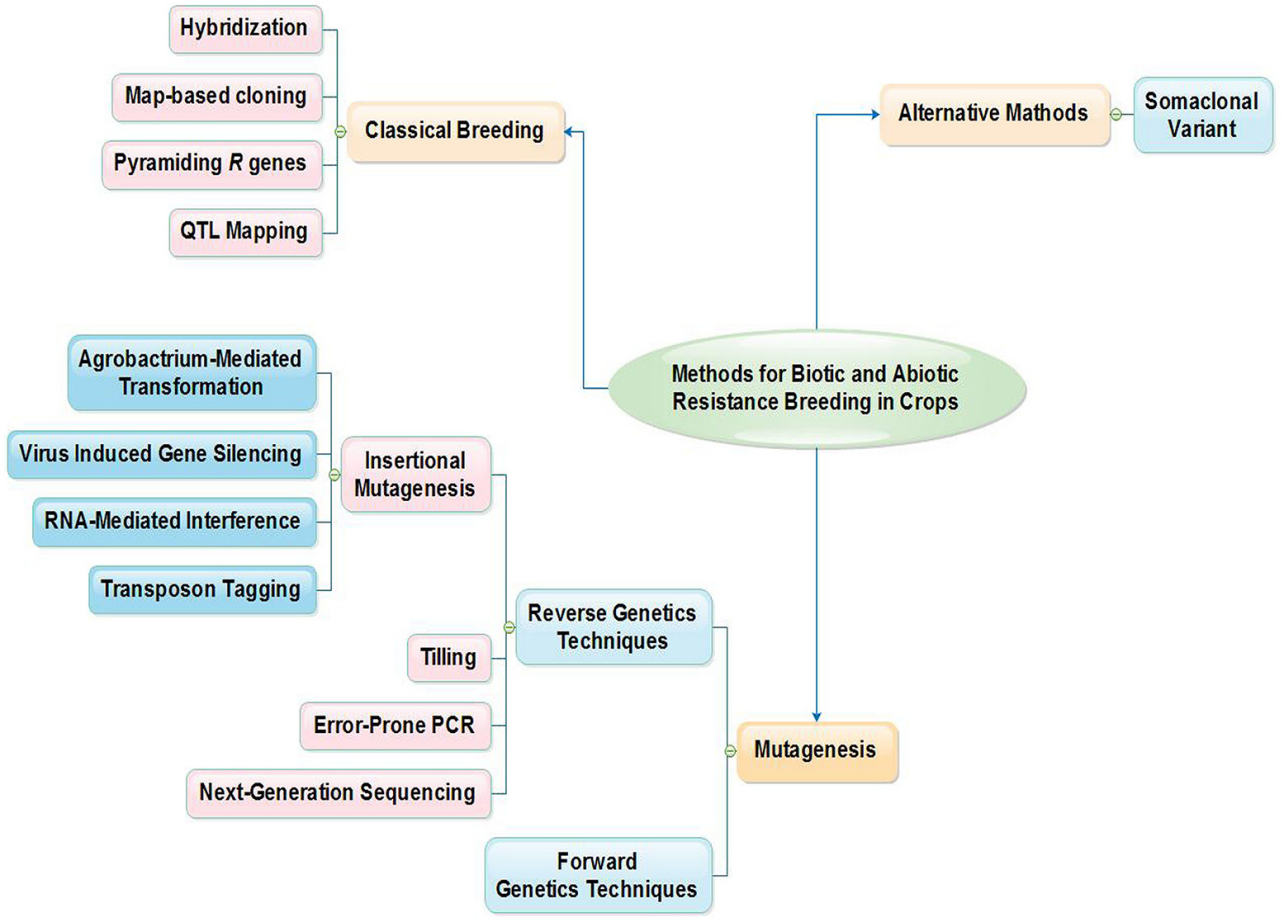
**Method for biotic and abiotic resistance breeding in crops**.

Biotic and abiotic stresses cause significant yield losses in food crop production and Improvement in stress tolerance of plant is a major breeding goal. Nowadays, different methods are being used to improve stress tolerance in plants (**Figure 1**). Diseases are among the most important limiting factors that affect rice production. More than 70 diseases caused by fungi, bacteria, viruses or nematodes have been reported on rice (Zhang et al., 2009). Rice blast (*Magnaporthe oryzae*) is the most devastating disease of rice because of its wide distribution and its destructiveness under conductive conditions (Skamnioti and Gurr, 2009; Helliwell and Yang, 2013; Helliwell et al., 2013). Among the biotic stresses blast disease is most important. Since there have been many blast disease outbreaks in rice, efforts have been made to develop new cultivars resistant to the blast disease. Earlier studies on the variability of this fungus relied mainly on the phenotypic characters and virulence test using a set of host differentials. These studies were only focused on screening and selection of rice varieties or advanced lines toward selected local blast pathotypes. Most of these phenotypic traits are highly variable as this pathogen is genetically unstable. Such kinds of studies are labor-intensive and time-consuming, require large greenhouse space and often lead to ambiguous results. Furthermore, they are influenced by environmental conditions, inoculation techniques and human errors during scoring (Shivayogi et al., 2002). Now strategic research concentrates on filling the gaps in the existing knowledge of biotic stresses on rice, especially improving molecular genetics of blast disease, with a view to develop an integrated management program for blast resistance. Over the past decades, we have seen the successful use of advanced molecular and genomic tools such as molecular markers, expressed sequence tags (ESTs), microarrays, and genetic transformations to explore the genetic basis of stress tolerance and eventually to develop crop cultivars improved for stress tolerance. The recent developments in DNA marker technology have helped to develop the concepts of QTLs mapping, marker-aided selection (MAS) and genetic transformation to produce plants of superior quality. In addition, molecular markers can be used for assessing genetic diversity, fingerprinting genotypes, separating hybrids from selfed progeny, and other uses. The actual identification of candidate DNA markers linked to resistance genes using fine mapping may well allow rice breeders to efficiently transfer these genes from donor cultivars into new, elite rice cultivars using marker-assisted selection (MAS). There is also a need to connect knowledge about genes and gene function to create new productive varieties that are a necessary element of a sustainable food supply for the future (Ashkani et al., 2015). Here we highlight a set of molecular tools that are currently being used to study the rice blast fungus. The information generated on recent methodology will help breeders to expedite breeding research in rice crops and explore a promising new concept which utilizes such molecular data to breed for durable resistance to rice blast.

## Rice And Rice Blast Importance

Rice (*Oryza sativa*), is the principal food for over half of the population of the world and supplies the main energy resource for almost 50% of the world’s population (Yu et al., 2002). In Asia, where 60% of the earths’ people live 90% of the world’s rice is grown and more than 3 billion Asians obtain 35–75% of their calories from rice and its products (Khush and Jena, 2009). Even though the world’s rice production increased from 257 million tons in 1966 to 600 million tons in 2000, the increase has not kept up with the demand for rice because of the corresponding increase in the human population during this time. It is estimated that rice production must increase by at least 40% in 2030 to meet ever-increasing demands (Khush, 2005). Hence, population increasing at an alarming rate, making the food security the major challenge in future.

Rice serves as an economically important crop and advances in molecular biology have made it a model monocot species among the cereal for genetics studies in breeding programs. Rice in comparison to other grass species has several attributes such as: small genome; extensive genetic resources; genetic transformation potential; synteny with other cereal genomes; comprehensive genetic and physical map of the genome; high density molecular map for gene mapping and map-based gene cloning; complete sequencing of the genome in indica and japonica rice cultivars; development of bacterial artificial chromosome (bac) and yeast artificial chromosome (YAC) libraries and development of the *Oryza* map alignment project (OMAP); and development of the genetic maps of chloroplast and mitochondrial genomes.

The study of homologies and diversities of markers and genes within and between species, genus or other taxonomic divisions is mentioned to comparative mapping (Paterson et al., 1991). This comparison involves analyzing the conserved area between maps of the order wherein markers occur; the conserved marker order is named ‘synteny.’ Comparative mapping may help in the construction of new linkage maps and the locations predictions of QTLs in varius mapping populations (Young, 1994). Infact, previous linkage maps may show an insight which markers are polymorphic and show an insight of linkage groups and the order of markers in the linkage groups. In the last few years, high-density molecular linkage maps of rice containing approximately 3000 markers have been developed making the marker density in the rice genome, on average, one marker per cM (Causse et al., 1994; Harushima et al., 1998; Lopez-Gerena, 2006).

**FIGURE 2:**
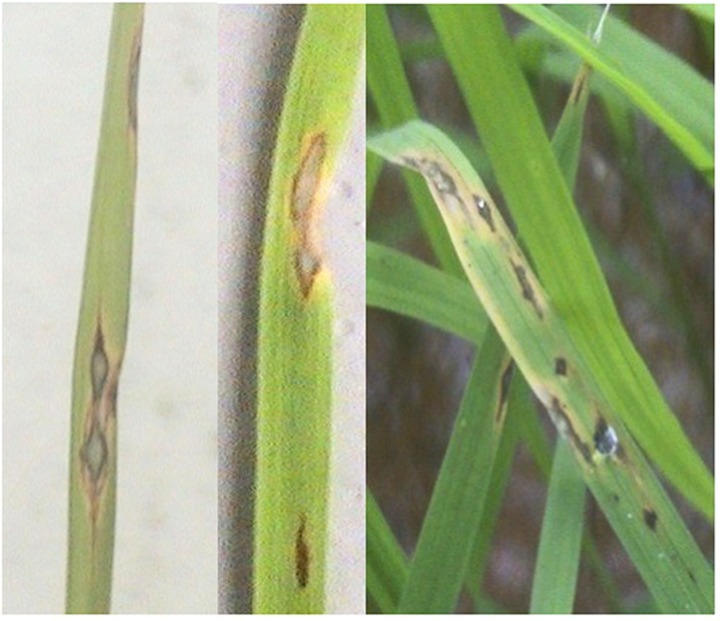
**Leaf blast disease symptoms.** Lesions are typically spindle-shaped; wide in the center and pointed toward either end. Large lesions usually develop a diamond shape with a grayish center and brown margin.

Rice blast is by far the most important disease that attack rice. The fungus *M. oryzae* = *M. grisae* (Cooke) Sacc [anamorph: *Pyricularia oryzae*], is the causal agent of blast disease (Couch and Kohn, 2002). The fungus colonizes leaves (leaf blast), panicles (panicle blast) and other parts of the rice plants, and causes huge crop loss in rice growing areas. Its most infections occur on the leaves and first symptoms of the disease appear as small brown or grayish dots on the leaves. After 2–3 days the dots develop to almost 1.5 cm long and 0.3–0.5 cm wide diamond-shaped lesions with a gray or white center (**Figure 2**), causing the infected leaves to die. The yield losses due to blast were reported to be between 30 and 50% in large rice producing areas under favorable environmental conditions (Correa-Victoria José and Zeigler, 1993; Skamnioti and Gurr, 2009). Efforts are underway to develop rice varieties with durable blast resistance. Therefore, continuous studies on blast disease are important in order to overcome this disease problem sustaining rice production in the future.

## Management Of Rice Blast And Efficient Ways For Crop Protection

Management of rice blast through the breeding of blast-resistant varieties is the most desirable means of managing blast, especially in developing countries. Rice cultivars with durable blast resistance have been recognized in several production systems. The deployment of rice cultivars with broad-spectrum resistance is practical means of controlling the fungal pathogen (Bonman, 1992; Bonman et al., 1992; Fukuoka et al., 2015). Rice blast control with resistant cultivars is much desired for farmers and consumers, because it can decrease fungicide application, subsequently reducing agrochemical pollution in the rice fields, thus reducing the cost of production. Local and wild varieties are normally used as sources for introgression of a new resistance gene into cultivated rice. Genetic resistance has been, and will continue to be, the major method of disease control of blast. The resistance in newly released rice cultivars to rice blast caused by *M. oryzae* can be lost due to the high level of instability in the genome of this fungus or due to the frequent breakdown of resistance under field conditions (Bonman, 1992; Bonman et al., 1992; Zeigler et al., 1994). One way to overcome this problem is through pyramiding of multiple *R* genes, each recognizing a unique set of *M. oryzae* isolates, into a single cultivar. Molecular markers techniques can be utilize in gene pyramiding in rice breeding programs to produce resistant cultivars and overcome to breakdown of disiease in early stage thereby sustaining rice yields and eventually to map based cloning of the gene. Such techniques as simpler method will save time and minimize costs especially for traits with laborious screening and it is more efficient to use of resources for plant breeding.

## Molecular Breeding Research To Improve Disease-Resistant Varieties

New rice varieties that combine higher yield potential with excellent grain quality, resistance to biotic and abiotic stresses, and input use efficiency are desperately needed. Diverse strategies for breeding durable resistance have been offered for rice blast. Some of these strategies, such as pyramiding (Bonman, 1992; Bonman et al., 1992), lineage exclusion (Zeigler et al., 1994), multilines (Abe, 2004) and mixtures (Zhu et al., 2000), are based on the use of complete and specific resistance genes. In general, in current agriculture three major strategies are used to improve disease resistance in crops. The first strategy involves improvement of cultural practices. The second approach comprises the improvement of crops through conventional or molecular marker-assisted breeding of disease resistant cultivars. The third strategy is the direct transformation of resistant genes into elite cultivars (Baulcombe, 2004). The use of molecular techniques for example the agrobacterium-mediated transformation allows the stable transfer of a transgene in a different variety or species, as well as to use a different promoter on a resistance-related gene to alter the intensity of gene expression (Baulcombe, 2004). To increase rice production and resistance, conventional rice breeding carried out during the last 50 years, resulted in the release of modern resistant varieties with high quality and yield. Despite, this method has played an important role in rice cultivar development over the past decades there are drawbacks as well. Conventional breeding progress is slow owing to several obstacles, such as: time-consuming and laborious selection process, difficulties in appropriate genotype selection due to the quantitative nature of most agronomic traits, several generations of crossing, selfing, and testing plants for resistance. In addition, traditional breeding is often negatively affected by linkage drag, which resulted in the transferring of loci conferring potentially undesired agronomic traits due to its close linkage with resistance loci. Recent advances in molecular genetics of rice have provided new tools for breeders to develop the rice varieties of the future which is known as molecular breeding. Only few years ago, the status of rice genetics was considered far behind that of other food crops such as maize, wheat, barley, and tomato. However, the last decade has seen a knowledge explosion in this area and rice is now considered a model plant for such research on cereal crops. Rice has been performed as a successful crop in biotechnology approaches leading to crop improvements. A vast reservoir of germplasm (>200,000 accessions) of both domestic and wild rice is available for genetic and breeding research. With the completion of rice genome sequence, many rice research now focused on functional characterization of rice genes, elucidation of the underlying mechanisms involved in major agronomic traits (e.g., high yield, grain quality, abiotic stress tolerance, and disease resistance), and subsequent translation of genomic knowledge into agricultural productivity via molecular breeding and improved cultural practice (Helliwell and Yang, 2013; Helliwell et al., 2013). Genetic studies of blast resistance in rice were established in Japan as early as 1917 (Ballini et al., 2008). To date, there are various molecular and biotechnological approaches to genetically improve rice crop for effective, durable and/or broad-spectrum resistance to major diseases. Currently, many resistance genes (*R*-genes) and QTLs in rice for blast have been identified and sequenced (Ashkani et al., 2014). These genes contribute to the understanding of the interaction between the disease and the host for breeding proposes. In addition, a wide variety of genes and mechanisms involved in rice defense response (e.g., pathogenesis-related proteins and other defense genes) have been identified and elucidated. Also, many molecular approaches including use of specialized promoters, modification of target protein structures have been studied and proposed to improve the effectiveness of transgenes (Baulcombe, 2004). During the past two decades, some rice research institute has been involved in the rice mutation breeding program to generate new varieties, in support of the crop breeding program. The main purposes of mutation breeding of rice have been improvement of agronomic traits, inducing resistance against diseases and pests, and enhancing the grain quality and grain taste. In the breeding program for rice in collaboration with IRRI (the Philippines) and JIRCAS (Japan) many modern varieties have also been released for commercial use. Traditional rice varieties have been widely used as genetic resources for biotic and abiotic traits of hybridization program. To complement conventional breeding method, molecular and transgenic method represents an increasingly important approach for genetic improvement of disease resistance and reduction of pesticide usage and various molecular strategies including use of specialized promoters, modification of target protein structures have been studied and proposed to improve the effectiveness of transgenes (Helliwell and Yang, 2013; Helliwell et al., 2013). MAS for quick indirect selection of the target gene by using molecular markers closely linked to a target gene as a molecular tag, quantitative trait locus (QTL) analysis and genetic transformation techniques are the most useful tools for rice molecular breeding especially to improve disease-resistant varieties. These techniques have been used to identify new germplasms and elite rice cultivars.

## Blast Disease Improvement Due To Molecular Markers Techniques

Molecular markers have played an increasing role in rice breeding for cultivar improvement, screening, selection and germplasm collections (Wang et al., 2007). The new sequencing tools provide valuable informations for the discovery, validation and assessment of genetic markers in populations (Sahebi et al., 2015). For instance, the analysis of next generation sequencing (NGS) data by means of bioinformatics developments allows discovering new genes and regulatory sequences and their positions, and makes available large collections of molecular markers (Perez-de-Castro et al., 2012). The whole genome sequence data substantially enhanced the efficiency of polymorphic marker development for QTL fine mapping and the identification of possible candidate genes (Wan et al., 2006). These performances can be useful as genetic resources for breeding of rice cultivars. The use of molecular markers for rice has been recently reviewed (Temnykh et al., 2001; Xu, 2002; Semagn et al., 2006; Lang et al., 2008; Kumar et al., 2009; Benali et al., 2011). In the case of rice blast (*M. oryzae*) a large number of the major genes had been identified and was targeted for mapping investigations using a variety of marker systems and approaches (Ashkani et al., 2014). DNA markers including : simple sequence repeats (SSRs), Single-nucleotide polymorphisms (SNPs) and small insertions/deletions (InDels), amplified fragment length polymorphisms (AFLPs), random amplified polymorphic DNAs (RAPDs), cleaved amplified polymorphic sequences (CAPS), and restriction fragment length polymorphisms (RFLPs) have been identified to be linked with blast resistance genes in rice (Ashkani et al., 2014; Tanweer et al., 2015). In recent year, scientists have used these markers for genetic mapping to identify candidate genes and QTLs in many plant species. Several genes of agronomic importance such as those that confer resistance to blast, bacterial leaf blight, brown planthopper, tungro and grassy stunt virus have been transferred from the wild species into the elite breeding lines of rice, including the quantitative trait loci (QTLs) for biotic and abiotic stress resistance (Amante-Bordeos et al., 1992; Brar and Khush, 1997).

DNA markers techniques provide us and rapid tool to select for the existence of multiple blast resistance genes without the need to test the progeny or inexact phenotypic disease screening (Fjellstrom et al., 2004). Through molecular markers tools many useful markers linked to the race-specific blast resistance genes (*Pi*-genes), has been identified and screened in segregating populations in rice (Fjellstrom et al., 2004; Sharma et al., 2005; Ashkani et al., 2011). PCR-based markers as example SSRs are precise, reliable and cost effective; this marker has been applied for the selection of plants containing blast resistance genes in rice at an early stage (Hittalmani et al., 2002; Ashkani et al., 2012). Microsatellites are SSR markers, and have been used extensively to identify genes and QTLs associated blast resistance in rice. Microsatellites are abundant in plants (McCouch et al., 2001), causing more polymorphism and better repetition over other marker systems. The genetic map covering all 12 rice chromosomes with at least one microsatellite at the distance of 0.5 cM has been developed by International Rice Microsatellite Initiative (IRMI; McCouch et al., 2002). Currently, breeders are focusing on MAS instead of using conventional breeding. Application of MAS reduces the time for phenotypic selection and saves the costs to select a desired trait (Koide et al., 2009). This method is helpful tool, and more accurate approach in introducing novel cultivars and it also help breeders to expedite breeding research in crops by enabling selection based on the genotype rather than on the phenotype. After the discovery of molecular markers linked with gene of interest, selection of specified traits to develop new cultivar could be made at an early level (Zhu et al., 2012). Pyramiding of linked genes into a single line or cultivar is one of the common applications of MAS.

Marker-assisted backcross breeding (MABC) as another technique recently has been given attention in rice breeding for the introgression of blast resistance genes (one or a few genes) into the susceptible or in an adapted or elite varieties. MABC is the process of using markers to select for target loci, minimize the length of the donor segment containing a target locus and accelerate the recovery of the recurrent parent (RP) genome during backcrossing (Charcosset, 1997; Hospital, 2001; Hasan et al., 2015). The main purpose of MABC is to transfer the desired character/or targeted gene along with recovering the recurrent parent characters/or genes. MABC is now playing an important role for the development of blast-resistant cultivars (Sundaram et al., 2009) and is superior to conventional backcrossing in precision and efficiency and time saving. Molecular markers which are tightly linked with important traits are used in MABC. Therefore, molecular markers are the tools that can be used to detect the presence of desire character in backcrossing and greatly increases the efficiency of selection. The methods and potential application of MAS and MABC for the Improvement of rice have been recently reviewed and described (Collard and Mackill, 2008; Hasan et al., 2015). Recently through application of MABC many blast resistance genes have been successfully introgressed into the genetic background and improved the blast resistance. Some successful examples for application of MAS and MABC in rice breeding programs aimed at improving blast resistance in this species are presented in **Table 1.**

**Table 1:** Examples for application of marker-assisted selection (MAS) and marker-assisted backcrossing (MABB) in rice.

Trait	Gene(s)/QTL(s)	Marker(s) used	Technique used	Application	Reference
Blast resistance	*Pi1, Piz-5, Pita*	RFLP	MAS	Pyramiding of three near isogenic lines (C101LAC, C101A51 and C101PKT) for blast resistance in into a single cultivar Co-39, each carrying the major genes *Pi1*, *Piz-5* and Pita, respectively	Hittalmani et al., 2000
Blast resistance	*Pi1*	SSR and ISSR	MAS	Applied for backcross breeding of variety (Zhenshan 97A)	Liu et al., 2002b
Bacterial blight Resistance + Blast resistance	*Xa21, Piz*	SSR	MAS	Functional for pyramiding of target traits	Narayanan et al., 2002
Blast resistance	*Pid1, Pib*, *Pita* and *Pi2*	SSR	MAS	*Pid1, Pib* and *Pita* genes were introduced into G46B cultivar, while *Pi2* Zhenshan97B cultivars of rice	Chen et al., 2004
Blast resistance	*Pi-z*	SSR	MAS	Closely linked with *Pi-z* locus has been successfully used for selection of blast resistance in a wide array of rice germplasm	Fjellstrom et al., 2006
Blast resistance + Bacterial blight resistance + Sheath blight resistance	*Xa13*, *Xa21*, *Pi54*, qSBR11	SSR [for blast resistance (*Xa13* and *Xa21)*, for bacterial blight resistance (*Pi54*), and Sheath blight resistance (qSBR11)]	MAS	MAS-assisted transfer of genes conferring the resistance toward three different diseases in rice	Singh et al., 2012a
Blast resistance + Bacterial blight resistance	*Pi*-genes, *Xa5*	SSR	MAS	Near-isogenic lines (NILs) derived from two blast resistant crosses (RD6 × P0489 and RD6 × Jao Hom Nin) were pyramided with IR62266 (*xa5*), to transfer bacterial leaf blight resistance to RD6 lines	Pinta et al., 2013
Blast resistance	*Pi-ta*	Gene specific marker	MAS	Existence of the *Pi-ta* gene in 141 rice germplasm has been successfully determined, but the results were more articulated when *Pi-ta* gene was introduced through advanced breeding lines	Wang et al., 2007
Submergence tolerance + Brown planthopper resistance + Blast resistance + Bacterial blight resistance	chr9 QTL, *Xa21*, Bph and QTLs blast, and quality loci	SSR and STS	MABB	MABB confirmed the transfer of gene and QTL for into cultivar KDML105	Toojinda et al., 2005
Blast resistance	*Pi1, Pi2, Pi33*	SSR	MABB	Introgressed into Jin23B cultivar through MABB	Chen et al., 2008
Blast resistance + Bacterial blight	*Pi1*, *Pi2*, *Xa23*	SSR [For blast resistance (*Pi1*, *Pi2*), for bacterial blight resistance (Xa23)	MABB	Sucessfully applied for breeding the variety (Rongfeng B)	Fu et al., 2012
Blast resistance	*Piz-5*, *Pi54*	SSR	MABB	Combination of blast resistance gene from donor lines (C101A51 and Tetep) into cultivar PRR78 to develop Pusa1602 (PRR78 + *Piz5*) and Pusa1603 (PRR78 + *Pi54*), respectively	Singh et al., 2012b
Blast resistance	*Pi-9(t)*	pB8	MABB	MABB applied to introgress the cultivar Luhui17	Wen and Gao, 2011
Blast resistance	*Pi-1, Pi-z*	SSR	MABB	Pyramiding of *Pi-1* and *Piz-5* genes into introduced PRR78 cultivars	Gouda et al., 2013


## Resistant Genes And Qtls For Blast Disease

Resistance to blast was classified into complete and partial resistance (Wang et al., 1994). Complete resistance is a qualitative character and race specific controlled by a major gene (*R* genes). Meanwhile, partial resistance is a quantitative character and non-race specific, which is controlled by many genes known as quantitative resistance loci (QRL; Young, 1996). However, if the resistance is highly partial, it can also be controlled by a major gene and is race specific. Qualitative and quantitative blast resistances have been reported in rice germplasm (Ou, 1985). Many qualitative resistance major genes (∼100 genes) for blast resistance have been identified and mapped in the rice genome (Sharma et al., 2012; Ashkani et al., 2014). About 22 *R*-genes have been successfully cloned and molecularly characterized. In the practice of resistance breeding, using a single R gene which has a broad resistance spectrum is more effective. There have been many reports on introgression of *Pi* genes related to blast disease into commercial and elite varieties. For example, the *Pi-9* gene that exists in the indica rice line 75-1-127 (Liu et al., 2002a), was introgressed from the wild species *O. minuta* (Amante-Bordeos et al., 1992). The *Pi-ta* allele was identified in *O. rufipogon* and *O. nivara*, or in their hybrids with *O. sativa* (Jena and Khush, 2000). These *R* genes function in a gene-for-gene fashion, so the pathogen can adapt by mutating or deleting the corresponding a-virulence gene. Therefore varieties those carrying R genes which confer high levels of resistance typically lose their resistance after a few years (Chen et al., 2003). Quantitative resistance donated by quantitative trait loci (QTL) are long-lasting disease resistance against a wide-range of pathogens, promising for sustainable rice production in the future (Song and Goodman, 2001). QTL mapping is a modern type of study to locate genes controlling a quantitative trait. Since the first publication of a QTL analysis of rice resistant to blast (Wang et al., 1994), several QTLs related to blast resistance have been detected using different type of markers, population and environment and have been published. We have summerized all these events in tabular form (**Table 2**).

**Table 2:** Quantitative trait loci (QTL), identified for rice blast resistance.

Mapping population	Parents used in crossing	Total No. of QTLs detected	Used markers	Reference
Recombinant Inbred Lines (RILs)	CT9993-5-10-1-m × KDML105 (F_8_); Zhenshan 97 × Minghui 63 (RILs); Moroberekan × Co39 (F_7_); Lemont × Teqing (F_8_); Lemont × Teqing (F_14_); Bala × Azucena (F_6_); Zhong 156 × Gumei 2 (F_8_); Oryzica Llanos 5 × Fanny (F_5_ and F_6_); SHZ-2 × Lijiangxin-tuan-heigu (LTH) (RILs); KDML105 × JHN (F_6_); Suweon365 × Chucheong (RILs)	186	RFLPs, SSR, RAPD, Isozymes, AFLPs, DR gene markers	Sirithunya et al., 2002; Chen et al., 2003; Wang et al., 1994; Tabien et al., 2002; Loan et al., 2003; Liu et al., 2004; Talukder et al., 2005; Wu et al., 2005; Lopez-Gerena, 2006; Noenplab et al., 2006; Cho et al., 2008;
Doubled Haploid (DH)	IR64 × Azucena; IR64 × Azucena; ZYQ8 × JX17	146	RFLPs, RAPD, Isozymes	Xu et al., 2004; Sallaud et al., 2003; Bagali et al., 2000
Single-segment substitution lines (SSSLs)	Developed by the use of HXJ74 as recipient and 24 accessions as donors	11	SSR	Zhang et al., 2012
Back cross population	Way Rarem × Oryzica Llanos 5 (IRGC 117017); *Oryza sativa* cv MR219 × *O. rufipogon* IRGC 105491; SHZ-2 × TXZ-13; *Oryzarufipogon* × cultivated rice IR64	45	SSR, SNP	Lestari et al., 2011; Rahim et al., 2012; Utani et al., 2008; Liu et al., 2011
F_2,_ F_3,_ and F_4_	Nipponbare × Owarihatamochi (F_4_ lines); Kahei × Koshihikari (F_2:3_); Tainung 69 × Koshihikari (F_2_); URN12 × Koshihikari (F_2_); Norin29 × Chubu32 (F_3_); Pongsu Seribu 2 × Mahsuri (F_2:3_); TAM × KHZ (F_2:3_); J*unambyeo* × *O. minuta* introgression line IR71033-121-15 (F_2:3_); *Danghang-Shali* × Hokkai 188 (F_2:3_)	60	RFLPs, SSR STS	Fukuoka and Okuno, 2001; Miyamoto et al., 2001; Sato et al., 2006; Zenbayashi et al., 2002; Ashkani et al., 2013a,b Nguyen et al., 2006; Rahman et al., 2011; Sabouri et al., 2011


Quantitative trait loci detection approach has been employed to map major or minor genes involved in the resistance (Wang et al., 1994; Fukuoka and Okuno, 2001; Miyamoto et al., 2001; Tabien et al., 2002; Zenbayashi et al., 2002; Chen et al., 2003; Sallaud et al., 2003; Talukder et al., 2004; Wu et al., 2005; Ashkani et al., 2013a,b). Identification of QTLs, associated with blast resistance has been delivered the effective genetics evidences for the molecular marker assisted breeding and cloning of the major genes. In the other word, QTL mapping is useful in identifying multiple loci controlling complete resistance in a highly resistant cultivar as well as in estimating the number, location and effect of genomic region involved in partial blast resistance (Sallaud et al., 2003). Many rice improvement programs now aim to incorporate quantitative or polygenic resistance into rice varieties. Previous studies have verified that genetic linkage maps constructed with various DNA markers are very useful for the analysis and detection of qualitative trait loci (Bao et al., 2000; Price et al., 2000). Molecular linkage maps have helped resolve the effects of minor and major QTLs and estimate the amount phenotypic variation explained at each locus. Molecular linkage maps have led to better understanding of genetic phenomena, such as interloci (epistasis) and intralocus (dominant) interactions (Grandillo and Tanksley, 1996), heterosis (Stuber et al., 1992) and identifying transgressive segregants (Tanksley, 1988, 1993).

## Gene Pyramiding For Blast Resistance

**FIGURE 3:**
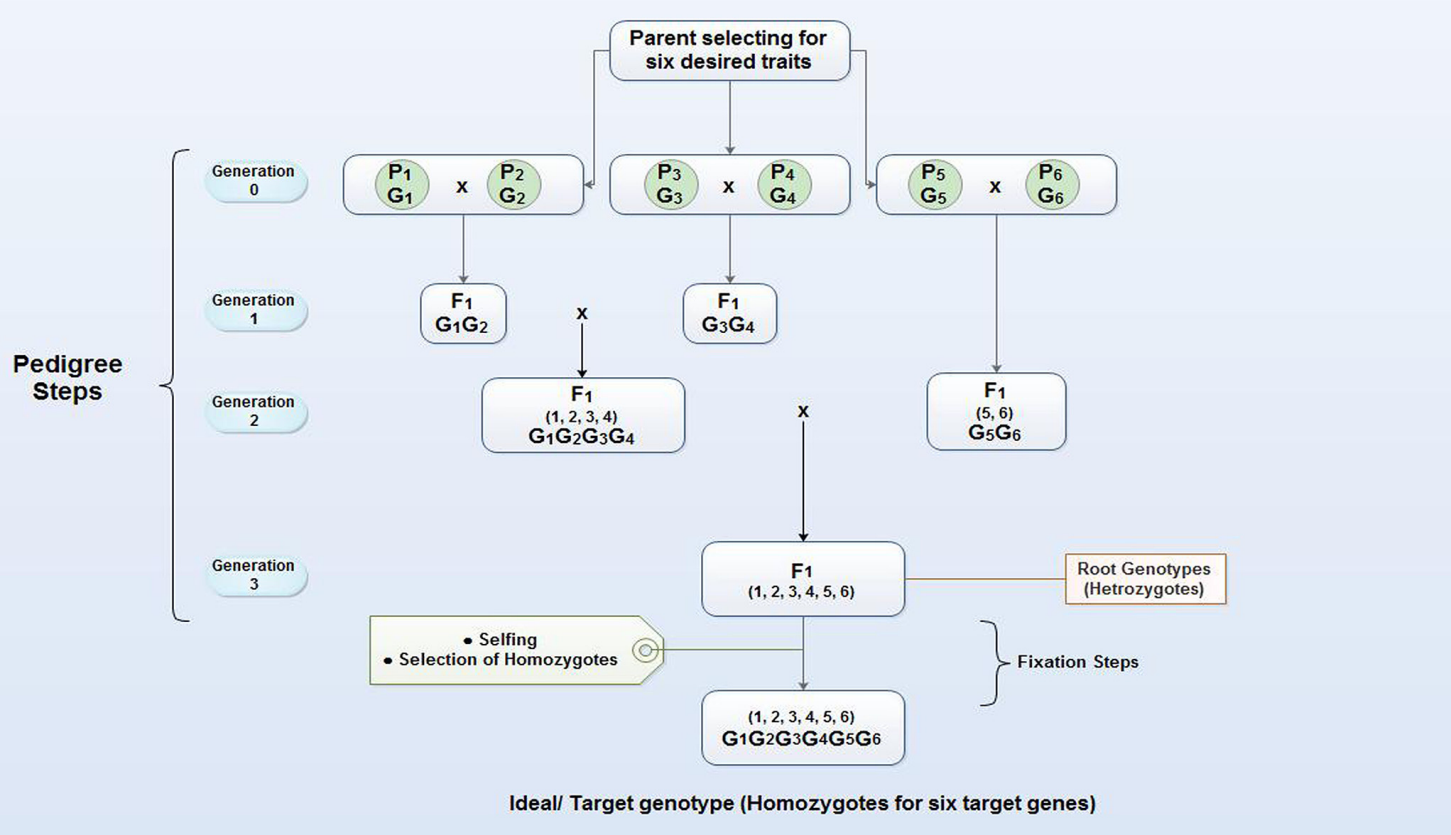
**Gene pyramiding scheme for cumulating six desired genes (G1–G6) which are present in 6 different parents or lines (P1–P6).** The gene pyramiding consists of two steps, pedigree, which aims at cumulating of all target genes in a single genotype (Root genotype) by crossing and selection; the second step is fixation which aims at fixing the target genes into a homozygous state (Ideal/target genotype).

Pyramiding is the accumulation of genes into a single line or cultivar. In a gene pyramiding, strategy is to cumulate genes identified in multiple parents into a single genotype (**Figure 3**). The end product of a gene-pyramiding program is a genotype with all of the target genes. Pyramiding multiple resistance genes provides durable stress resistance expression in crops. Gene Pyramiding technique broadly is used for combining multiple disease or pest resistance genes for specific races of a pathogen or insect to develop durable resistance. It helps in crop improvement program and reduces breeding duration. Different R-genes often confer resistance to different isolates, races or biotypes. Combining their resistance broadens the number of races or isolates that a more than one character in a variety at the same time. Developing elite breeding lines and varieties often requires plant breeders to combine desirable traits from multiple parental lines, particularly in the case of disease resistance. Gene pyramiding can be accelerated by using molecular markers to identify and select plants that contain the desired allele combination in very early stage, resulting in obvious savings of resources including greenhouse or field space, water, and fertilizer. Therefore, marker technology can help existing plant breeding programs and allows researchers to access, transfer and combine genes at a rate and with a precision not previously possible. MAS based gene pyramiding could facilitate in pyramiding of genes effectively into a single genetic background (Joshi and Nayak, 2010). Factors such as the number of genes to be transferred, the distance between the target genes and flanking markers calculated in genetic mapping studies, the number of genotype selected in each breeding generation and the nature of germplasm is critical for successful gene pyramiding program. Gene pyramiding is considered one of the most effective strategies for achieving durable resistance against blast disease in rice (Shinoda et al., 1971; Hittalmani et al., 2000; Koide et al., 2010) and have successfully used for accumulating different blast resistance genes in elite rice cultivars (**Table 3**).

**Table 3:** Example of gene pyramiding for blast resistance trait in rice.

Traits	Parental lines	Pyramided genes	DNA marker(s) used	Reference
Blast resistance	C101LAC, C101A51	*Pi1, Pi2* and *Pi33*	SSR	Chen et al., 2008
Blast resistance	IR5, IR8, IR20, IR22, IR24, IR26, IR28, IR29, IR30, IR32, IR34, IR36, IR38, IR40, IR42, IR43, IR44, IR45, IR46, IR48, IR50 IR52, IR54, IR56, IR58, IR60, IR62, IR64, IR65, IR66, IR68, IR70, IR72, IR74	*Pib* and *Pita*	SSR	Fujita et al., 2009
Blast resistance	CO39	*Pish* and *Pib*	SSR	Koide et al., 2010
Blast resistance	IR64, JHN	Multiple resistance QTLs	SSR	Sreewongchai et al., 2010
Blast resistance	Rongfeng B	*Pi1*, *Pi2 Xa23*	SSR	Fu et al., 2012
Blast resistance	Jin 23B	*Pi1, Pi2*, and *D12*	SSR	Jiang et al., 2012
Blast resistance	C101LAC, C101A51	*Pi-1* and *Pi-2*	RG64 and C481	Mahdian and Shahsavari, 2013
Blast and bacterial leaf blight resistance	RD6 × P0489; RD6 × JHN	Four QTLs for blast resistance and one gene for bacterial leaf blight (xa5)	SSR	Pinta et al., 2013
Blast resistance	C101A51, Tetep	*Piz5* and *Pi54*	SSR	Singh et al., 2013
Blast resistance	Carnaroli, Baldo, Arborio	*Piz* and *Pi5*	SSR	Urso et al., 2013
Leaf blast resistance	Koshihikari	*Pi21*, *Pi34*, and *Pi35*	SSR	Yasuda et al., 2014
Blast resistance	GZ63-4S	*Pi2* and *Xa23*	SSR (*M-Xa23)*	Jiang et al., 2015


## Allele Mining And Blast Resistance Genes

Allele mining is the commonly used approach to identify novel alleles or allelic variants of a gene/or candidate genes of interest, based on the available information about the genes, from a wide range of germplasm. This technique possesses good potential to be used in molecular plant breeding of crop improvement programs. The success of allele mining mainly depends on the type of genetic materials used for screening and should be as diverse as possible and availability of genome and gene sequence information of a particular crop species. For efficient allele mining, wild relatives and local landraces are used because they are reservoirs of useful alleles hidden in their phenotype (Tanksley et al., 1996). The current availability of complete rice genome sequences in addition to several bioinformatic tools have made it possible to mine allelic diversity throughout rice germplasm. EcoTilling and sequence based allele mining are the two widely used approaches in allele mining. Compared to EcoTilling, sequence based allele mining strategy is reported to be simpler and cost effective approach (Ramkumar et al., 2010; Ashkani et al., 2015). Allele mining possesses wide range of applications within crop improvement among them are, allele identification, allelic variation characterization, haplotypes identification, analysis of haplotypes diversity among different haplotypes of the same gene or among the related haplotypes, evolutionary relationship, similarity analysis and development of molecular markers to differentiate a particular allele from other alleles. To date allele mining strategy has been well demonstrated by many researchers. Allele mining of genes from wild and cultivated rice species aims to detect superior alleles for blast resistance (Kumari et al., 2013). So far, mining approaches have been used to identify novel and superior alleles of many major blast resistance genes from different cultivated rice varieties and wild species (**Table 4**). Through allele mining techniques functional marker to differentiate the resistance and susceptible alleles of *Pi54* has been developed (Ramkumar et al., 2011). Costanzo and Jia (2010) analyzed the sequence level similarity for *Pikm* alleles, derived from 15 different rice cultivars. *M. oryza* has also been differentiated from *M. grisea* by using allele mining (Couch and Kohn, 2002).

**Table 4:** Summary of allele mining report for blast resistance genes.

*R-Genes/Locus*	Chromosome	Rice germplasm	Reference
*Pi-ta*	12	From wild rice species [*O. rufipogon* (Griff) and from *O. rufipogon* (ETOR)]	Yang et al., 2007; Geng et al., 2008
*Pi-ta*	12	From *O. rufipogon*	Huang et al., 2008
*Pi-ta*	12	From cultivated (AA) and wild species and invasive weedy rice	Lee et al., 2009, 2011
*Pi-ta*	12	In 26 accessions, consisting of wild rice (*O. rufipogon*), cultivated rice (*O. sativa*) and related wild rice species (*O. meridionalis* and *O. officinalis*) collected from ten different countries of the world	Yoshida and Miyashita, 2009
*Pi-ta*	12	From landraces and wild *Oryza* species	Ramkumar et al., 2010
*Pi-ta*	12	In Indian land races of rice	Sharma et al., 2010
*Pi-ta*	12	From Indian landraces of rice collected from different ecogeographical regions including the northwestern Himalayan region of India	Thakur et al., 2013a
*Pi-kh (Pi54)*	11	From wild and cultivated species of rice	Rai et al., 2011
*Pi-kh (Pi54)*	11	From the blast-resistant wild species of rice, *O. rhizomatis*	Das et al., 2012
*Pi-kh (Pi54)*	11	From six cultivated rice lines and eight wild rice species	Kumari et al., 2013
*Pi-kh (Pi54)*	11	In Indian land races of rice	Sharma et al., 2010
*Pi-z(t)*	06	In Indian land races of rice	Sharma et al., 2010
*Piz(t)*	06	In 529 land races of rice collected at three different geographical locations of India	Thakur et al., 2013b
*Pid3*	06	From 36 accessions of wild rice *O. rufipogon*	Shang et al., 2009; Xu et al., 2014
*Pid3-A4*	06	From wild rice A4 (*O. rufipogon*)	Lv et al., 2013
*Pi9*		In different rice species, five AA genome *Oryza* species including two cultivated rice species (*O. sativa* and *O. glaberrima*) and three wild rice species (*O. nivara*, *O. rufipogon*, and *O. barthii*).	Liu et al., 2011
*AC134922*	11	Rice lines from various sources	Wang et al., 2014


## Conclusion

Disease management extremely needed to sustain the world for food consumption. Rice blast caused by *M. oryzae* is the most severe fungal disease, which limits the rice production and causing the yield loss of 157 million tons of rice per annum in the worldwide (Kaundal et al., 2006). Development of resistant varieties with durable resistance by incorporating new genes into the improved germplasm has been proved to be economical, environmentally friendly and effective to control the rice blast disease (Skamnioti and Gurr, 2009). The availability of different molecular tools allows characterization of genes of interest and identification of plants carrying the target genes and might well serves to improve the efficiency of conventional breeding. Due to molecular dissection it is now possible to identify blast resistance genes and QTLs or combined effects of multiple loci with major and minor effects. The marker developed from these genes or QTLs can be used in marker assisted selection for selection of resistance without confounding the effects of environmental factors. DNA markers that co-segregate with the gene are a powerful method for use in crop protection and can be routinely employed in various aspects of plant genome analysis such as genetics and plant breeding. Information provided on genetics of blast resistance of local traditional variety is very useful for rice resistance breeding program in every country. Recent molecular breeding strategy such as gene pyramiding and allele mining holds greater prospects to attain durable resistance against biotic and abiotic stresses in crops. Identification of novel and superior resistance alleles of the blast resistance genes is an important task in the rice breeding program. The novel alleles are very useful in breeding programs and can be utilized gainfully to develop productive and superior plants.

## Future Perspectives And Consideration

The major difficulty in controlling rice blast is the durability of genetic resistance. Enhancing the host plant resistance is being considered as the best approach to handle the rice blast disease. Rice cultivars containing only a single *R* gene to a specific pathogen race often become susceptible over time due to the emergence of new virulent races. Understanding of genetic identity of contemporary *M. oryzae* is important for accurate deployment of rice cultivars with different *R* genes. Stacking *R* genes with overlapped resistance spectra can lead to long lasting resistance. For combinations of different blast resistance genes in host plant in the rice blast breeding programs, superior alleles of the targeted genes should be considered. During the evolution and artificial selection processes, a significant portion of beneficial alleles have been left behind in the landraces and wild species (McCouch et al., 2007), which can be used for the development of better rice varieties. Effective blast management also requires international cooperation. The knowledge gained by collaborative effort ought to lead to more effective methods to reduce crop loss due to blast disease worldwide. Although considerable progress has been made toward understanding the nature of disease resistance genes, defense responses, and the signal transduction leading to activation of defense responses in rice, the whole story is still far from clear. Studies of the molecular biology of disease resistance will be helpful in improving rice varieties for high production for increasing population. The completion of the rice genome project and availability of structural genomic data for the public can undoubtedly accelerate research on the molecular biology of rice disease resistance. Development of new molecular techniques, methodologies such as functional geonomics and DNA microarrays for global analysis of gene expression should urge rice breeders to integrate these techniques to conventional breeding. Rice research should more focus on identifying more durably resistant genes, tagging of these genes with molecular markers and pyramiding these genes or QTLs through molecular MAS. Monogenic resistance to blast is less stable but varieties with pyramided monogenes or QTLs are durably resistant. Molecular breeding strategy can help in the introduction of durably blast-resistant rice cultivars thereby sustaining rice yields. Candidate gene identification through rice functional genomics has great potential for developing more durably resistant varieties.

## Conflict of Interest Statement

The authors declare that the research was conducted in the absence of any commercial or financial relationships that could be construed as a potential conflict of interest.
